# Antimicrobial properties of the novel bacterial isolate *Paenibacilllus* sp. SMB1 from a halo-alkaline lake in India

**DOI:** 10.1038/s41598-019-47879-x

**Published:** 2019-08-09

**Authors:** Harjodh Singh, Manpreet Kaur, Manoj Jangra, Sunita Mishra, Hemraj Nandanwar, Anil Kumar Pinnaka

**Affiliations:** 1 Academy of Scientific and Innovative Research, (AcSIR), CSIR Campus, Chennai, India; 2 Council of Scientific and Industrial Research (CSIR) - Central Scientific Instruments Organisation, Sector 30C, Chandigarh, 160030 India; 3 Clinical Microbiology & Bioactive Screening Laboratory, Council of Scientific & Industrial Research -Institute of Microbial Technology, Sector -39A, Chandigarh, India; 4 MTCC-Microbial Type Culture Collection & Gene Bank, CSIR-Institute of Microbial Technology, Chandigarh, 160036 India

**Keywords:** Applied microbiology, Antimicrobials

## Abstract

Antibiotic-resistance is ever growing burden on our society for the past many years. Many synthetic chemistry approaches and rational drug-design have been unable to pace up and tackle this problem. Natural resources, more specifically, the microbial diversity, on the other hand, make a traditional and still the best platform to search for new chemical scaffolds and compounds. Here, we report the antimicrobial characteristics of novel bacterial isolate from a salt lake in India. We screened the bacterial isolates for their inhibitory activity against indicator bacteria and found that four novel species were able to prevent the growth of test strains studied *in vitro*. Further, we characterized one novel species (SMB1^T^ = SL4-2) using polyphasic taxonomic approaches and also purified the active ingredient from this bacterium. We successfully characterized the antimicrobial compound using mass spectroscopy and amino acid analysis. We also allocated two novel biosynthetic gene clusters for putative bacteriocins and one novel non-ribosomal peptide gene cluster in its whole genome. We concluded that the strain SMB1^T^ belonged to the genus *Paenibacilllus* with the pairwise sequence similarity of 98.67% with *Paenibacillus tarimensis* DSM 19409^T^ and we proposed the name *Paenibacillus sambharensis* sp. nov. The type strain is SMB1^T^ (=MTCC 12884 = KCTC 33895^T^).

## Introduction

Increasing burden of antibiotic resistance around the globe is of grave concern. The present antibiotics are almost ineffective to fight against the deadly infections caused by many bacteria^1^. It is estimated that till 2050, around 10 million people may die from antimicrobial-resistant infections if current scenario persists. Presently, the methods and measures taken globally to tackle this problem are insufficient and slow^2^. Various strategies such as high throughput screening of synthetic chemical compound libraries and determination of new targets with the help of genomic studies were not successful in finding potential antimicrobial entities^3^.

In recent years, natural products have gained significant attention to overcome the gap in drug discovery and development due to their structure versatility and potential biological activity^4–8^. Majority of the antibiotics consumed at present are either the natural compounds or derivatives thereof, which were discovered from soil *Actinomycetes* in 1940–1960s i.e. golden era of antibiotics. The soil has been extensively mined since then for the new antimicrobial molecules and now seems exhausted. Therefore, looking for alternative microbial sources or unique niches of microbes would be an asset to find novel antimicrobial compounds.

Biodiversity of halophilic bacteria holds a huge potential to produce new and unexplored antimicrobial entities. Tonima Kamat *et al*. reported the antimicrobial activities demonstrated by the halophilic bacteria isolated from salt pans^9^. Similarly, Toktham *et al*. also reported the effectiveness of the halophiles as antimicrobials^10,11^. Recently, Atirah *et al*. discussed the purification of a bacteriocin from *Halomonas* sp.^12^. Intracellular proteins of *Virgibacillus marismortui* and *Terribacillus halophilus*, i.e., glucanase and chinatase respectively have been reported for their antimicrobial activity^13^. Based on these observations and since there are not many reports available from India showing the antimicrobial potential of halophiles, we studied the biodiversity of halophilic bacteria isolated from Sambhar Lake in Rajasthan, India. In the present study, we screened the bacterial isolates from this lake for their antimicrobial activity. We have characterized one novel species strain SMB1 in this manuscript. Moreover, we purified and characterized the antimicrobial compound from the fermentation broth. Tandem mass spectroscopy, amino acid analysis, and whole genome data were used to identify this antimicrobial compound. Additionally, we predicted two novel bacteriocin gene clusters and one non-ribosomal peptide gene cluster in the whole genome of this novel species. The novel species SMB1^T^ was characterized using polyphasic taxonomic approaches.

## Results

### Antimicrobial screening of bacterial strains

More than hundred bacterial strains were isolated from the Sambar Lake and screened for their antimicrobial activity against *E. coli* (MTCC 1610)*, Staphylococcus aureus* (ATCC 25923)*, Bacillus subtilis* (ATCC 6633), and *Candida albicans* (MTCC 224). The cell-free supernatant and/or crude fermentation extract of fifteen isolates inhibited the growth of Gram-positive bacteria. These positive isolates were identified based on their 16S rRNA gene sequences. They comprised of four novel species among them as shown in Table 1. Our group recently described three of these novel species viz; SMB4^T ^^14^, AK73^T ^^15^ and AK74^T ^^16^. In the current work, we characterized SMB1^T^ as novel species and studied its antimicrobial activity.

**Table 1:** Screening results for the production of antimicrobial compounds.

S.No	Strain	Closest homolog	24 h Supernatant		48 h Supernatant		48 h Extract	
			ATCC-25923	ATCC-6633	ATCC-25923	ATCC-6633	ATCC-25923	ATCC-6633
1	A 17	*Halomonas salifodinea*	−	−	−	−	**+**	−
2	A9	*Halomonas mongoliensis*	−	−	−	−	−	**+**
3	8b	*Pseudomonas stutzeri*	−	−	−	−	**+**	−
4	SL 12-4	*Bacillus licheniformis*	**+**	−	**+**	−	**+**	−
5	8a	*Paracoccus aestuarii*	−	−	−	−	**+**	−
6	**SMB1** ^**T**^	***Paenibacillus tarimensis***	−	−	−	−	**+**	**+**
7	7a	*Halomonas salfodinea*	−	−	−	−	**+**	−
8	SL 6–1	*Halomonas salifodinae*	−	−	**+**	−	**+**	−
9	**SMB-4** ^**T**^	Salibacterium nitratireducens*	−	−	**+**	**+**	−	−
10	**AK74** ^**T**^	Bacillus lacus*	−	**+**	−	**+**	**+**	**+**
11	**AK73** ^**T**^	Bacillus alkalilacus*	−	−	−	−	**+**	**+**
12	SL4-1R	*Alkalibacterium pelagium*	−	−	−	−	−	**+**
13	SL6-1R	*Halomonas salifodinae*	−	−	−	−	**+**	**+**
14	SL5-1R	*Bacillus sonorensis*	−	−	−	−	**+**	**+**
15	SL6-3R	*Halomonas salifodinae*	−	−	−	−	**+**	−

ATCC-25923- *Staphylococcus aureus;* ATCC-6633 *- Bacillus subtilis*.

*These strains are already described as novel species by our group previously.

^+^no growth of indicator strain around the well; ^−^ growth of indictor strain around well.

### Identification and characterization of the antimicrobial compound from strain SMB1^T^

**Figure 1:**
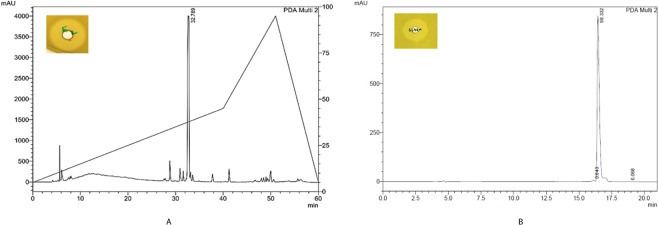
(**A**) Profile of the cation exchange active peak of the antimicrobial compound of SMB1^T^. The activity of the peak is inserted in the picture; (**B**) HPLC profile of the purified compound of SMB1^T^. The activity of the peak is inserted in the picture.

**Figure 2:**
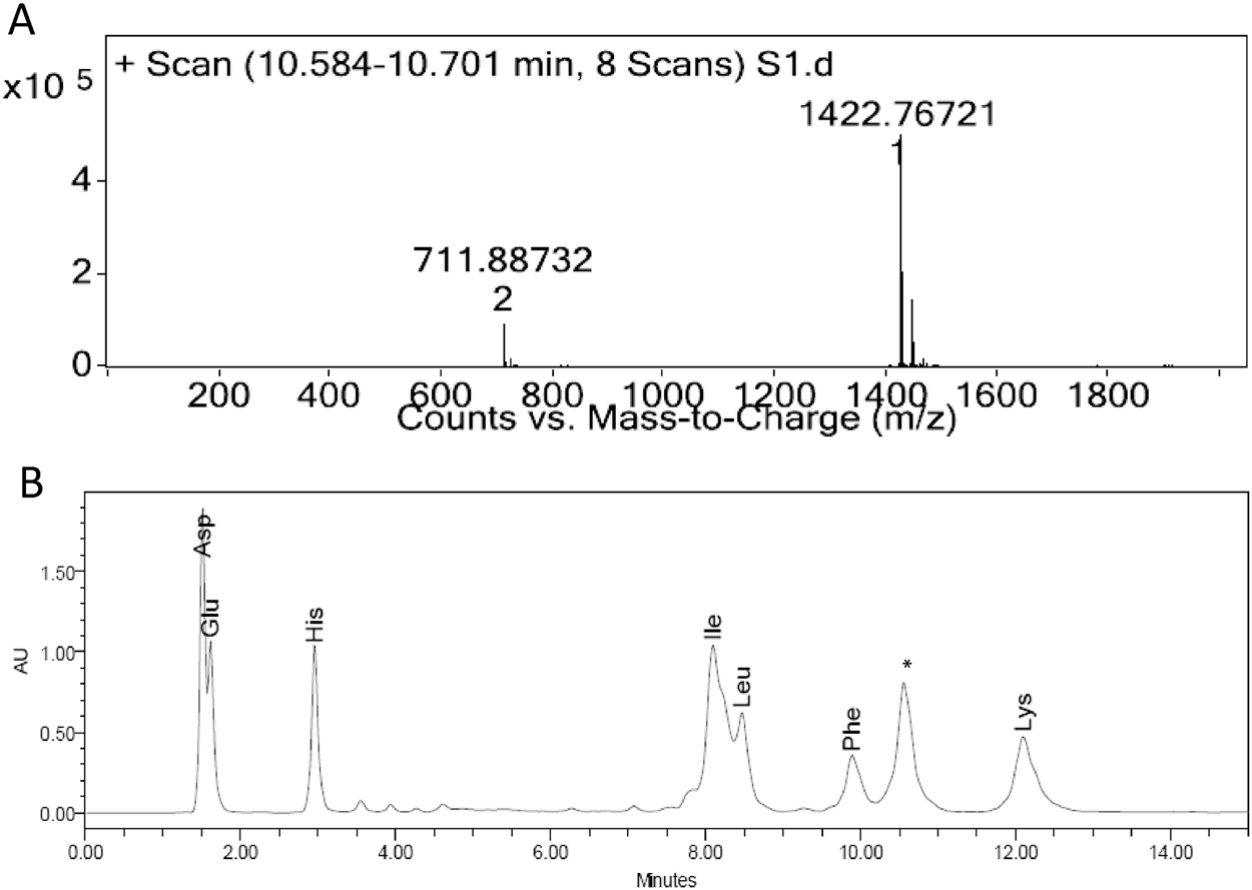
(**A**) LCMS profile of active fraction of antimicrobial compound of SMB1^T^; (**B**) Amino Acid chromatogram of the pure antimicrobial compound of SMB1^T^.

**Figure 3:**
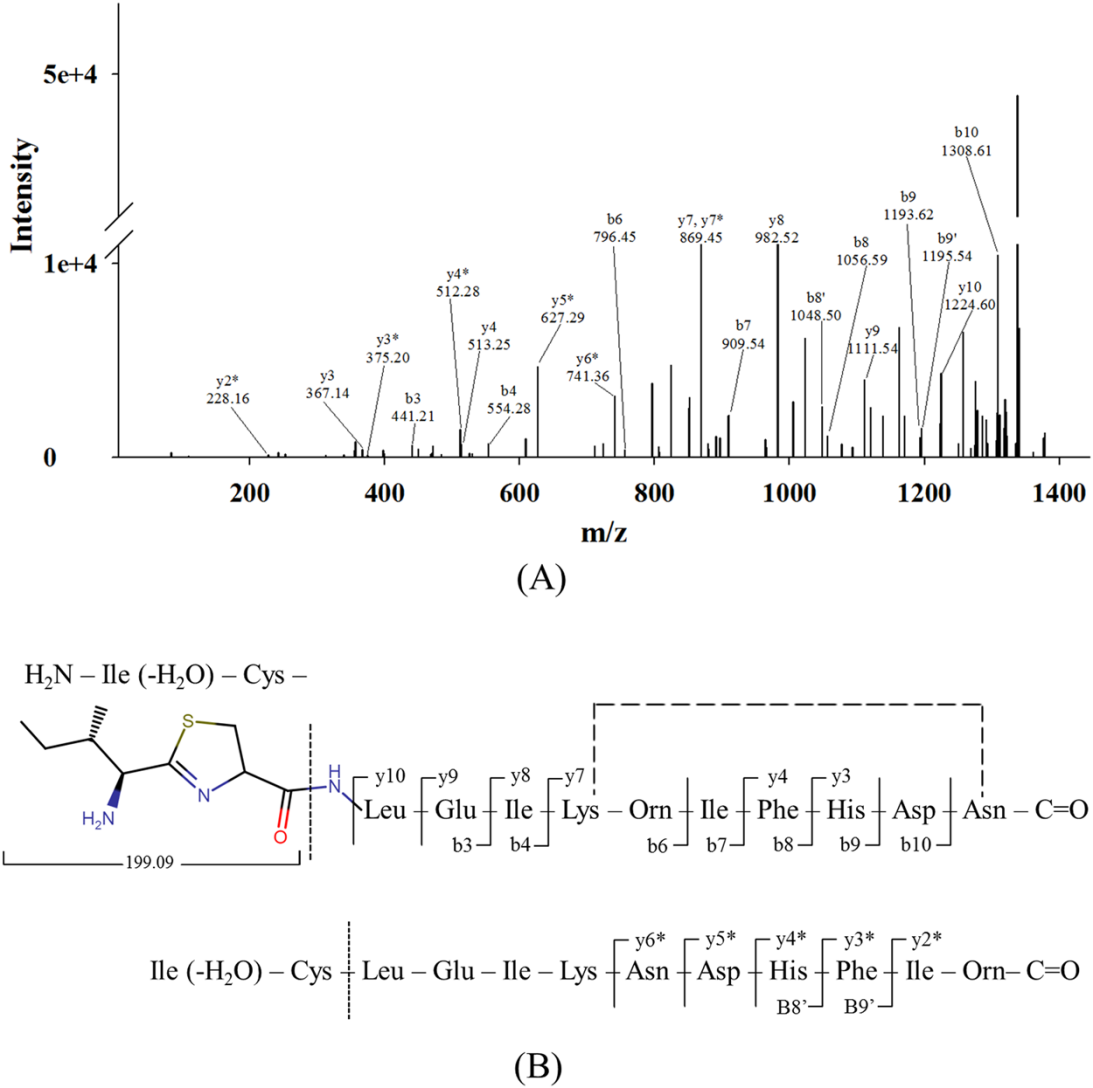
MSMS Spectrum of isolated compound. (**A**) Assignment of b and y ions; (**B**) Amino acid sequence deduced from the MSMS data. Both α-carbonyl group and ɛ-amino group of the lysine residue at position 6 are involved in amide linkage with ornithine (position 7) and asparagine (position 12) respectively, to form a cyclic heptapeptide.

**Figure 4:**
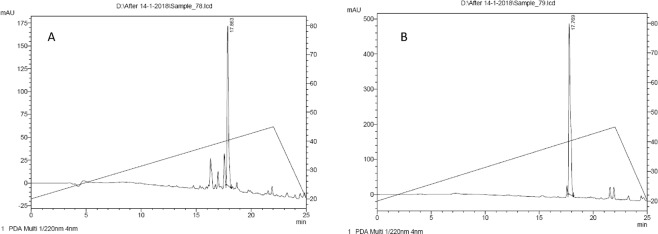
(**A**) HPLC profile of standard Bacitracin A; (**B**) HPLC profile of purified compound from SMB1^T^.

The strain SMB1^T^ showed consistent antagonistic activity against Gram-positive bacteria. Partial purification of the antimicrobial compound was achieved through cation-exchange chromatography. The active fraction was eluted at 0.5 M NaCl concentration. Further, the purification of the active compound was performed on RP-HPLC. Figure 1A shows the HPLC chromatogram of cation-active dialysate. The active compound eluted at RT = 32.7 min. The purity of the compound was more than 95% (Fig. 1B). The pure compound showed m/z value at 1422.76 [M + H]^+^ in LC-ESI-MS and one doubly charged ion at 711.88 [M + 2H]^+2^ was also formed as illustrated in Fig. 2A. The molecular mass of the compound was deduced as 1421.75 Da. Tandem MS and amino acid analysis were further carried out to identify the compound. In the amino acid analysis, we found the presence of aspartic acid, glutamic acid, histidine, isoleucine, leucine, phenylalanine, lysine and one non-standard amino acid which later was identified as ornithine Fig. 2B. MS-MS data also supported the amino acid composition. Mass spectroscopy and amino acid profile revealed that the active compound belongs to bacitracin family and its molecular mass was similar to that of bacitracin A (C_66_H_103_N_17_O_16_S, calc. mass 1421.749 Da). The MS/MS data was consistent with what we observed for standard bacitracin A (Alfa Aeser, Thermofisher Scientific, India) (Fig. S1). We studied the MS/MS data extensively and annotated all the b and y ions present in the raw spectrum (Fig. 3A). Bacitracin A is a cyclic peptide containing twelve amino acid residues^17,18^. The lysine at position 6 is involved in double linkage with ornithine at position 7 using α- carbonyl group while with asparagine at position 12 using ε-amino group. In tandem mass spectroscopy, such residues are most vulnerable and thus we observed two series of b and y ions upon linearization of the peptide after the breakage of either of the bonds. Each bond leads to a different primary sequence and we were able to assign both series of ions as can be seen in Fig. 3B. For further confirmation, we performed the comparative analysis of the antimicrobial compound produced by the SMB1^T^ strain with standard bacitracin A. As shown in Fig. 4A,B, both compounds had significantly similar retention time in analytical RP-HPLC. Moreover, the antimicrobial activity spectrum against selected indicator strains was also similar (Fig. S2). This data collectively suggested that compound isolated in this study is bacitracin A.

### Whole genome analysis and putative biosynthetic gene clusters

**Figure 5:**
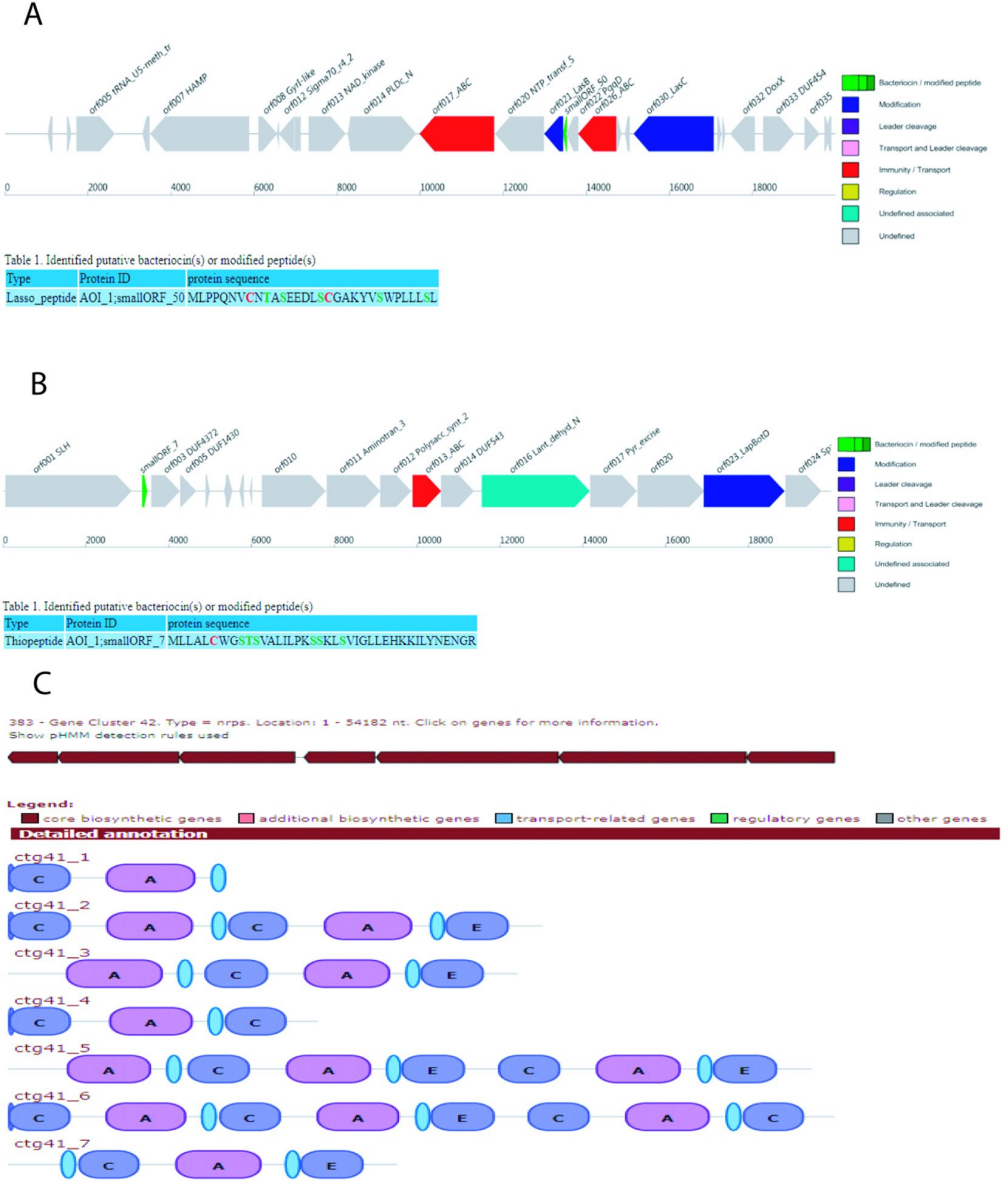
Antimicrobial biosynthetic gene cluster in the genome sequence of strain SMB1^T^ (**A**) Cluster 1, lassopeptide predicted using BAGEL3; (**B**) Cluster 2, thiopeptide, a putative bacteriocin predicted using BAGEL3; (C) Cluster 3, non-ribosomal peptide predicted using anti-SMASH.

The size of the genome of the strain SMB1^T^ is 5661449 bp with 4943914 total reads (N50 size 247161, L50 8 and N75 176162). This sequence was the draft genome of the strain SMB1^T^ in which 63 contigs were obtained. We predicted genes from the ABySS assembled contigs using Glimmer^19^. We found 5,282 genes in the assembly. The G + C content as predicted in the genome analysis was 53.0%, this falls in the G + C content range i.e 45–54% generally found in the genus *Paenibacillus*^20^. The assembled fasta file was uploaded on Rapid Annotation using Subsystem Technology (RAST) tool. This is a fully automated system for genome annotations. For further confirmation of the production of bacitracin by the strain SMB1^T^, we looked for the genes encoding for the synthesis of the bacitracin, we were able to locate the partial cluster at contig 383 showing the presence of Bacitracin synthetase 3 (BA3) gene as shown in Fig. S3. This enzyme complex encodes for the amino acids that are required for the synthesis of bacitracin. Hence, with this annotation results we confirmed that our strain SMB1^T^ is having the genes for the synthesis of bacitracin which was purified in the present study. Moreover, we subjected the whole genome sequence to other tools like as anti-SMASH (Antibiotics & Secondary Metabolites Analysis Shell) and BAGEL to find out the genes associated with the antimicrobials and the secondary metabolites. Two novel biosynthetic gene clusters were obtained in the BAGEL analysis (Fig. 5A,B). The gene clusters encoding for the lasso-peptide (cluster1) and thiopeptide (cluster2), the putative bacteriocins were identified using BAGEL 3. Another novel biosynthetic gene cluster was identified using anti-SMASH; it belonged to non-ribosomal peptide (NRP) secondary metabolite, and was located on contig 41 as shown in Fig. 5C. The software predicted thirteen adenylation domains and six epimerase domains. This analysis suggests that the predicted peptide contains thirteen amino acid residues; six out of them may be D-amino acids. The gene cluster exhibited less than 40% sequence identity with its closest homologue from other bacterial species. This indicated that the compound encoded by this cluster may be a novel compound and needs further investigation. The other clusters also displayed low levels of homology with other peptide gene clusters. The comparison of bacteriocin and NRPS gene cluster with their homologs are given in the Supplementary Material (Supplementary Figs S4 and S5). The genome sequence of the strain SMB1^T^ has been deposited in the GenBank database and Accession Number QKRB00000000 was obtained.

### Characterization of novel species SMB1^T^

**Figure 6:**
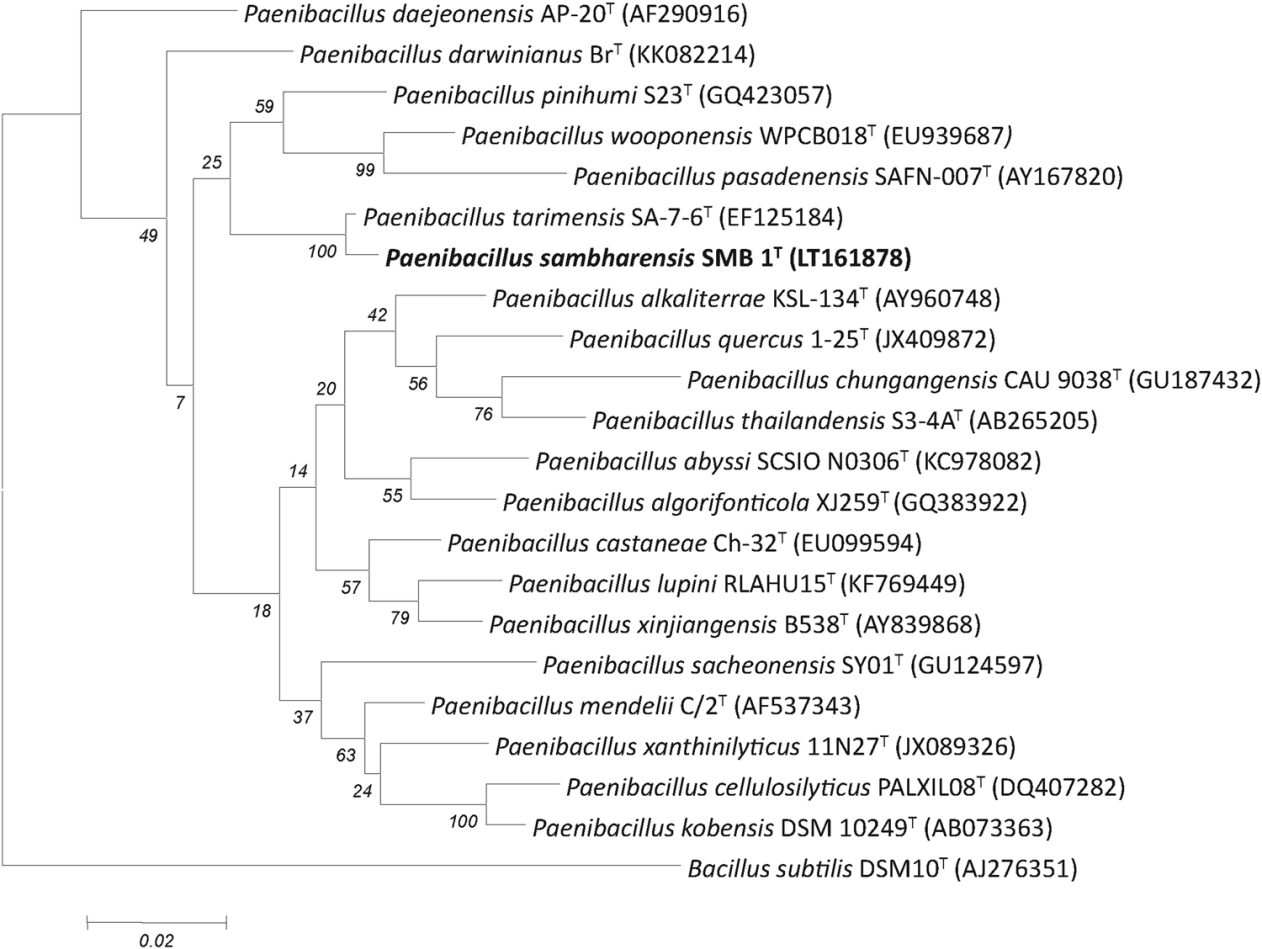
Neighbor-joining phylogenetic tree, based on 16S rRNA gene sequences, showing relationships between different strains of the genus *Paenibacillus* spp. *Bacillus subtilis* was taken as the outgroup. Bar, 0.02 substitutions per nucleotide position.

The strain SMB1^T^ was characterized using polyphasic taxonomic approach. The 16S rRNA (Accession Number LT161878) gene was showing 98.6% sequence similarity with *Paenibacillus tarimensis*. The sequence similarity with other members of the genus *Paenibacillus* is between 92.71% and 95.32%. The phylogenetic tree analysis demonstrated that the strain SMB1^T^ belongs to the genus *Paenibacillus* and its closest homolog is *Paenibacillus tarimensis* (Fig. 6). The strain SMB1^T^ is non-motile, straight rod-shaped Gram-positive bacterium with dimension 0.43–0.69 µm wide × 2.25–4.18 µm long (Fig. S6). The colonies were irregular with raised elevation, diameter 2–3 mm, cream color on ZMA plates, whereas after 48 hours in same conditions the colonies tend to appear reddish in color. The spore formation was noticed after 48 h.

The strain SMB1^T^ was able to grow between 30°C to 42°C and had an optimum temperature for growth at 37°C. The pH range for the growth was from pH 6.0 to 9.0, with the optimum growth at pH 7.0. Optimum growth occurred at salinities from 2% (NaCl, w/v) and the salinity range that the strain can withstand is 0–3%(NaCl, w/v). The phenotypic characteristics of the strain SMB1^T^ in comparison to its closely related species are described in Table 2. The results observed using phenotypic fingerprinting (VITEK 2 GP) are represented in Table S1.The fatty acid profile (Table S2) revealed the presence of branched and saturated like C16:0 (18.17%), C17:0 (4.06%), iso-C15:0 (4.17%), iso-C16:0 (5.83%), iso-C17:0 (5.09%), anteiso-C15:0 (48.86%) and anteiso-C17:0 (13.82%). Hydroxy fatty acids were absent. Overall fatty acid profile of strain SMB1^T^ was same as those of the strain DSM-19409^T^ and however, the saturated fatty acids C17:0 and branched fatty acids iso-C17: 0 was absent in DSM-19409^T^. Fatty acids C14: 0, C16: 1 *ω11c* and iso-C14: 0 was absent in strain SMB1^T^ but present in DSM-19409^T^. Hence, it clearly demonstrates the difference in their fatty acid profiles. The DNA base composition of strain SMB1^T^ was 54 mol% G + C (*Tm*). According to the DNA-DNA hybridization, the relative binding ratio with *Paenibacillus tarimensis*, DSM 19409^T^ was 46.45% (results are obtained from the average of the triplicates)*. The relation was found to be significantly less in comparison to the threshold value for the species delineation i.e. 70%*^21^. These results demonstrate the distinction of the strain SMB1^T^ from its closest neighbor. The digital protologue of this strain has been registered on their website (http://imedea.uib-csic.es/dprotologue/) under the taxonumber TA00612.

**Table 2:** Features that distinguish strain *Paenibacillus sambharensis* SMB1^T^ from the closely related species of the genus *Paenibacillus tarimensis* DSM-19409^T^.

Characteristics	*Paenibacillus sambharensis* SMB 1^T^	*Paenibacillus tarimensis* DSM-19409^T^
Cell size	2.25–4.18 × 0.43–0.69	3.0–6.0 × 0.5–0.8 µm
Motility	−	+
Flagella	−	+
Endospore	+	+
Colony color	Cream red	cream
Salinity growth range (%)	0–3	0–3
Salinity Optimum (%)	2	2
Temperature growth range (°C)	30–42	25–45
Optimum growth temperature (°C)	37	37
pH growth range (Optimum)	6–9(7)	6–9(7.5)
Oxygen requirement	+	+
**Biochemical:**		
Catalase	+	+
Phenylalanine deamination	+	w
Methyl red	−	+
Voges Proskauer’s	−	+
Indole	−	−
H_2_S	−	−
Oxidase	−	−
**Hydrolysis of:**		
Aesculin	+	+
Gelatin	−	−
ONPG	−	−
Tween 80	+	−
Urea	−	−
**Utilization of:**		
Fructose	−	−
Trehalose	+	+
Xylose	+	+
L-Arabinose	+	w
Raffinose	−	−
Melibiose	+	+
Sucrose	−	−
Mannitol	−	−
Citrate	−	−
Lactose	−	−
Mannose	+	+
Cellobiose	+	+
Adonitol	−	−
Rhamnose	+	−
Melizitose	+	w
N-acetyl-D-glucosamine	−	−
Xylitol	−	−
D-Arabinose	−	w
Malonate	−	−
**Antibiotic susceptibility (µg/disc):**		
Tetracycline (30)	S	S
Neomycin (30)	S	S
Cefazolin (30)	I	S
Penicillin G (2 units)	S	R
Gentamycin (10)	S	S
Kanamycin (30)	S	S
Polymixin B (300)	I	S
Cefprozil (30)	I	S
Amoxycillin (30)	S	I
Cephanroxcil (30)	S	S
Lincomycin (2)	S	R
Cefalexin (30)	S	R
Chloraphenicol (30)	S	S
Vancomycin (30)	I	S
Novobiocin (30)	I	I
Chlortetracycline (30)	I	R
DNA G + C content (mol%)	54.0	53.7

+, Positive; −, negative; w, weakly positive; R, resistant; S, sensitive; I, moderately sensitive.

## Discussion

Sambhar Lake is the one of the largest Salt Lake in India. It has the extreme hypersaline environment. These extreme conditions harbor microorganisms with valuable and distinct characteristics. The extremophiles survive the harsh and hyper environmental conditions and produce unique and uncommon bioactive molecules and secondary metabolites. These compounds are industrially stable and have many biotechnological applications^22^. The main objective of our study was to explore the diversity of the Sambhar Lake for the screening of the antimicrobial compounds. We isolated hundred bacterial strains from the lake samples and screened all of them for their antimicrobial activities using agar well diffusion assay. Fifteen isolates showed inhibitory activity against the indicator strains. The 16S rRNA gene sequencing data revealed that these isolates included four novel bacterial species, having pairwise similarity percentage less than or equal to 98.5%. We recently published three out of four strains as novel species. In the present work, we studied the strain SMB1^T^ for the purification and identification of the antimicrobial compound and also characterized it as a novel species. This strain had a pairwise sequence similarity of 98.67% with *Paenibacillus tarimensis* DSM 19409^T^ while the sequence similarity with other members of this genus was between 92.71% and 95.32%. So, we performed polyphasic taxonomic characterization to describe the strain. Different species of genus *Paenibacillus* were isolated and characterized till date from various ecological niches such as soils, plants, animals, polar Antarctic habitats, alkaline environments, marine sources or cold and desert environments^23–26^. The phylogenetic analysis based on the 16S rRNA gene sequences revealed that the strain SMB1^T^ is closely related to the *Paenibacillus tarimensis* DSM 19409^T^ and they shared the same clade. The genus *Paenibacillus* is reported to have antesio-C15:0 as major cellular fatty acids^20^, likewise our results showed the major fatty of anteiso-C15:0 (48.86%) in case of SMB1^T^ and anteiso-C15:0 (61.69%) for *Paenibacillus tarimensis* DSM 19409^T^. Major differences were also observed in the cellular fatty acids, as unsaturated fatty acids were absent in strain SMB1^T^, but they were present in the strain DSM 19409^T^ (C16: 1 *ω11c)*. Similarly, fatty acids C17:0 and iso-C17: 0 were present in our strain while absent in DSM 19409^T^. Moreover, the DNA-DNA hybridization results showed the relative binding percentage was below 70%. Hence, it clearly supports that the strain SMB1^T^ is the novel species. Based upon the phenotypic and genotypic analyses, we concluded the strain SMB1^T^ belongs to the novel species of genus *Paenibacilllus* and thus we proposed the name *Paenibacillus sambharensis* sp. nov for this strain (sam.bhar.en’sis. N.L. masc. adj. *sambharensis* pertaining to Sambhar Lake). The type strain is SMB1^T^ (=MTCC 12884^T^ = KCTC 33895^T^).

The *Paenibacillus* genus has been studied widely for producing a diversity of secondary metabolites, including enzymes, exopolysaccharides, and antimicrobial peptides and other industrially important bioactive molecules^27^. Polymyxins, which are active against Gram negatives and fusaricidins, the antifungal peptide are the best examples of antibiotic products of the *Paenibacillus* genus^27^. *Paenibacillus* also produces bacteriocins, for example, *P. polymyxa* NRRL B-30509 produces paenicidin^28^ and *Paenibacillus* sp. strain A3 produces penisin^29^. In the present research work, we have identified and characterized an antimicrobial peptide from our strain SMB1^T^. The whole genome analysis identified three novel biosynthetic gene clusters in this strain. We presumed that one of these clusters might be responsible for the observed antimicrobial activity. Hence we purified and characterized the antimicrobial compound from the fermentation broth. Using MS, MS/MS and amino analysis we confirmed that the compound is bacitracin A. Bioactivity was also found to be similar when assessed at the similar concentration, in comparison to standard bacitracin A. Though we were not able to obtain the complete biosynthetic cluster for the antimicrobial peptide bacitracin, but we found that the contig 383 of the genome sequence contains the genes encoding for the bacitracin synthesis. Recently, a draft genome sequence of a *Paenibacillus polymyxa* strain also revealed bacitracin biosynthetic gene cluster^30^. This further validates that the strain SMB1^T^ produces antimicrobial peptide bacitracin A. Bacitracin is a polypeptide known to be produced by *Bacillus subtilis* and *Bacillus licheniformis*^31^. Apart from this, we also found novel biosynthetic clusters for the putative bacteriocin i.e. lassopeptide and thiopeptide. Thiopeptides and lasso peptides are known for their antimicrobial activities^32^. In a recent report, *Paenibacillus dendritiformis* C454 was reported to produce novel lasso peptide paeninodin^33^. Additionally, we also identified one biosynthetic gene cluster for the non-ribosomally synthezized peptide. This suggests that the strain SMB1^T^ holds the potential as antimicrobial producing species. To the best of our knowledge, this is the first report describing the production and purification of the antimicrobial compound bacitracin A from the genus *Paenibacillus*. Moreover, the other novel species isolated from salt lake also showed antimicrobial activity and are deposited in a public repository. These strains could be explored in future for the isolation of bioactive compounds. Additionally, the novel biosynthetic gene cluster found in the whole genome of strain SMB1^T^ could be heterologously expressed and checked for their antimicrobial activity. This approach has been used in several antimicrobial clusters, for example malacidins^34^. These BGCs are cryptic gene clusters and alternatively, they may be expressed when placed under strong inducible promoters. As explained in the report by, Zipperer A *et al*. 2016, the compound lugdunin was not initially produced by the strain *Staphylococcus lugdunensis* in the fermentation broth, so they expressed the biosynthetic gene cluster by adding the strong promoter to produce the strain in liquid broth^8^. This approach can also be done in case of these BGCs as they are showing similarity less than 40% to the already known BGCs. Overall, this research demonstrates that novel species harbored from extreme niche hold potential to produce antimicrobial compounds.

## Materials and Methods

### Isolation of bacterial strains and antimicrobial screening

Sediment and water samples were collected in the sterile 50 ml polypropylene tubes (Tarsons, India) from different sites at Sambhar Lake, Rajasthan **(**GPS coordinates 26°55.520′N 075°11.827′E). The pH at different sampling sites was 8–12 and the temperature was 28 °C- 35 °C. Serially diluted samples were plated on different media such as Zobell marine agar (ZMA), Reasoner’s 2 A Agar (R2A agar) with 2% NaCl (w/v), modified Zobell marine agar containing NaCl (2–10% w/v) and pH range from 7–10 for the isolation of various halophilic bacteria. Optimization for salinity, pH, and temperature was done to check the optimum growth parameters. The pH was adjusted to 8.0–10 with the Na_2_CO_3_ solution (20%, w/v) and incubated at 30 °C and 37 °C for 3–7 days. The plates were monitored regularly and each unique colony was purified and preserved in 20% glycerol stock at −80 °C. The isolates were screened for their antimicrobial activity using agar well diffusion assay against *Staphylococcus aureus* ATCC 25923, *Bacillus subtilis* ATCC 6633 (Equivalent MTCC 441), *E. coli* MTCC 1610 and *Candida albicans* MTCC 224. Two to three colonies were inoculated in Zobell marine broth and incubated at 37 °C for 24–48 h. The cultures were harvested by centrifugation after 48 h, and the crude fermentation extracts using Diaion HP20 resins were prepared as described in the next section. The cell-free supernatants (100 µl) at 24 h and 48 h along with the crude extracts were loaded on seeded agar plates containing the indicator strain. The plates were incubated for 12–24 h and zones of inhibition were observed. The sterile medium without inoculation of culture was extracted in the similar way and served as negative control. Positive isolates having inhibitory activity were identified using 16S rRNA gene sequencing. Genomic DNA was extracted using DNA isolation kit (Zymo Research, California, D6005) and 16S rRNA gene was amplified. The sequencing was performed with the Genetic Analyzer ABI 3130XL (Applied Biosystems, California, USA). The sequence obtained was analyzed using EzTaxon sequence based database (https://www.ezbiocloud.net).

### Purification of the antimicrobial compound from the strain SMB1^T^

*Paenibacillus* sp. SMB1^T^ was grown in 700 ml ZMB in 2 L flask at 37°C and 180 rpm. After 36 h, the culture was harvested by centrifugation at 12,000 × g for 15 min. Subsequently, the cell-free supernatant was incubated with Diaion HP-20 (Supelco, Sigma-Aldrich, USA) resins (2% w/v) for 3 h. The resins were washed with 10% methanol and the bound components were eluted with 100% methanol. The solvent was evaporated under vacuum (Rotary evaporator BUCHI R-300). The crude extract was re-dissolved in Milli-Q. The antimicrobial compound was partially purified by cation-exchange chromatography (SP Sepharose, 10 mM ammonium acetate, pH 5.0). Bioactivity-guided fractionation was performed and the active fractions were pooled. The cation-active fraction was dialyzed using the 0.5–1 kDa membrane. Final purification of the antimicrobial compounds was carried out by high-performance liquid chromatography (HPLC) (SHIMADZU with PDA detector, XBridge Waters column, C18, 5 µm, 10 × 250 mm). The mobile phase consisted of solvent A, 5 mM ammonium acetate buffer (pH 5.5) and solvent B, 100% acetonitrile. The gradient elution was performed as 5–45% solvent B in 40 min, 45–90% B in 12 min and reverse 90–5% B in 8 min. The flow rate was kept at 3.0 ml/min. 500 µl sample was injected and the peaks were analyzed at 220 nm. All peaks were collected and assayed for bioactivity. The active peak was identified and purity was determined by analytical HPLC. Antimicrobial activity of the purified compound was checked against *Staphylococcus aureus* ATCC 25923.

### Mass spectrometry and amino acid analysis

The purified compound was subjected to LC-ESI-MS (Agilent 6550 *I* funnel QTOF) in positive ion mode. The mass spectrum was analyzed in the range of 400–4000 m/z. For MALDI-TOF analysis, the sample was mixed with α-cyano-4-hydroxycinnamic acid (CHCA) matrix and mass spectrum was obtained on MALDI-TOF mass spectrometer (AB Sciex 5800). MS/MS analysis was performed on the same instrument with TOF-TOF analyzer. The amino acid analysis was carried out with PICO-TAG amino analysis system (Waters) as per the manufacturer’s instructions.

### Comparative analysis of antimicrobial compound with standard bacitracin A

Bacitracin A was purchased from Alfa Aeser, Thermofisher Scientific, India. MS and MS/MS analysis of bacitracin A were performed in a similar way as described for antimicrobial compound isolated in this study. The MS/MS spectrum of isolated compound was compared with bacitracin A data and manually annotated in details. Also, the retention time of both compounds was compared in analytical HPLC under the similar conditions. Bacitracin A and isolated compound were dissolved at the same concentration (0.5 mg/ml) and their antimicrobial activity was checked against *Micrococcus luteus* and *Staphylococcus aureus*.

### Whole genome sequencing and bioinformatics analysis

Genomic DNA was isolated from the strain SMB1^T^ and the whole genome sequencing was performed on Illumina HiSeq sequencing platform using paired end library. Around 4–5 Mb data was obtained. *De Novo* assembly was performed using Spades, MaSuRCa, ABySS, and Velvet. We used ABySS assembly for all further downstream analysis since it had better statistics than all other assemblies generated^35^. 63 contigs were obtained with the ABySS assembly. The functional analysis was performed using Rapid Annotation using Subsystems Technology (RAST) version 2.0^36^. BAGEL3^37^ and anti-SMASH^38^ were used to predict the biosynthetic gene clusters for secondary metabolites and antimicrobial peptides. Default search parameters were used in antiSMASH and BAGEL3 mining.

### Characterization of SMB1^T^ strain

Sequence similarity search of SMB1^T^ strain indicated that *Paenibacillus tarimensis* and *Paenibacillus lacus* were the closest phylogenetic neighbors, with a pair-wise sequence similarity of 98.6%, 95.5% respectively. Thus, the strain SMB1^T^ was characterized in comparison to its closest type strain *Paenibacillus tarimensis* DSM 19409^T^. The phylogenetic tree was constructed using the neighbor-joining method in the MEGA6 software.

#### Morphological characterization

The strains SMB1^T^ and DSM 19409^T^ were grown in Zobell Marine Agar medium at 37 °C for 48 h. The shape, size, color, margin, and elevation of the colonies were observed. The cell shape was observed using phase contrast microscopy at 1000X magnification (BX51; Olympus, Japan). The cell size was measured using the transmission electron microscopy. The cells were grown in the Zobell Marine broth and the bacterial pellet was washed with PBS (pH 7.4). 10 µl of the sample was loaded on the copper carbon-coated grids and kept undisturbed for 10 minutes (300mesh), (Polysciences, Inc. USA cat #24933–25). After drying, 2% PTA (phosphotungstic acid) at pH 6 was added for 3 minutes and the grid was dried and observed under TEM (JEOL JEM-2100; Camera, ES500W Model 782).

#### Physiological characterization

KB003:Hi25^TM^ Carbohydrate Identification Kit and KB009 (HiMedia Laboratories, India) were used for other biochemical tests. Strains SMB1^T^ and DSM 19409^T^ were grown in Zobell Marine Agar medium until the log phase; the turbidity was adjusted to 0.1 OD at 620 nm. The specified wells in the kit were inoculated with 50 µl of the samples and the strips were incubated at 37 °C for 18–24 hours. The reagents were added in the selective wells as per the manufacturer’s instructions^39^. The change in color was observed and results were recorded.

#### Effect of temperature, pH and NaCl concentrations on the growth

Growth at varying temperature conditions (10, 15, 25, 30, 37, 38, 42, 50 and 55 °C) was measured with the Zobell marine agar plates streaked with the strains. To check the effect of pH the strains were inoculated in ZMA at pH 5.0, 5.7, 6.8, 8.0, 9.0 and 11.0 adjusted with different buffer systems such as; 0.1 M citric acid/0.1 M sodium citrate pH 4.0–5.0, 0.1 M KH_2_PO_4_/0.1 M NaOH pH 6.0–8.0, 0.1 M NaHCO_3_/0.1 M Na_2_CO_3_ (pH 9.0–10.0). Growth at various NaCl concentrations (0, 0.5, 1, 2, 4, 6, 8, 9, 10, 12 and 14% (w/v)) was monitored as described previously^40^.

#### Phenotypic fingerprinting using VITEK -2

VITEK^®^2 system was used for the phenotypic fingerprinting of the cultures SMB1^T^ and DSM 19409^T^. The cultures were analyzed based on their metabolic activities for the utilization of various nitrogen, carbon, and other nutrient sources. VITEK 2 is the automated system, it contains 64 welled VITEK^®^2 GN cards (France) having different substrates in each well. The bacterial cell culture (diluted in 0.45% (w/v) of NaCl) with the OD 0.5 measured by DensiCheck meter (bioMe’rieux) was used in an automated sampling system the cards were incubated in the in-build incubator in VITEK 2 machine. The results were recorded.

#### Antibiotics susceptibility assay

The susceptibility of strain SMB1^T^ and the DSM 19409^T^ to different antibiotics was checked using disc diffusion assay as per Clinical and Laboratory Standards Institute guidelines^41^. The antibiotics discs (Hi-Media, India) used were Tetracycline (30 mcg), Neomycin (30), Penicillin G (2 units), Gentamycin (10 mcg), Kanamycin (30 mcg), Amoxycillin (30 mcg), Cephanroxcil (30 mcg), Lincomycin (2 mcg), Cefalexin (30 mcg), Chloramphenicol (30 mcg), Cefazolin (30 mcg), Polymixin B (300 units), Cefprozil (30), Vancomycin (30 mcg), Novobiocin (30 mcg) and Chlortetracycline (30 mcg).

#### Fatty acid methyl ester (FAMEs) analysis

The strains SMB1^T^ and DSM 19409^T^ were grown till the logarithmic phase. Cellular fatty acid methyl esters (FAMEs) were obtained according to manufacturer’s protocol. The samples containing the FAMEs were subjected to GC (6890) and the different fatty acids were separated and analyzed with the Sherlock Microbial Identification System (MIDI-6890 with database TSBA6)^42^.

#### DNA-DNA Hybridisation

The G + C content of genomic DNA of the strain SMB-1^T^ and the type strain was determined spectrophotometrically (lambda 35, Perkin Elmer, Waltham, MA, USA) by using thermal denaturation method^43^. For DNA-DNA Hybridisation genomic DNA was isolated for both the strains. The fluorimetry method was used to measure the relative binding ratio of the samples. Step One Plus Real-Time PCR system (Applied Biosystems) was used with the thermal cycler in 96-well plate as explained by^44^. 2X SSC buffer was used for dissolving the DNA. SYBR Green in the ratio 1:10000 was used for the detection of the binding. The program used for the experiment was denaturation at 95 °C for 10 min, the re-association temperature was 74.4 °C for 10 sec (240 cycles) and holding stage 25 °C for 5 min. The florescence readings were recorded and the relative binding percentage was calculated according to the method reported by^45,46^.

### Accession numbers

16S rRNA gene sequence was submitted to GenBank/EMBL/DDBJ under Accession Number LT161878. The genome sequence of the strain SMB1^T^ has been deposited in the GenBank/NCBI database and Accession Number QKRB00000000 was obtained.

### Ethical approval

This article does not contain any studies with human participants or animals performed by any of the authors.

This manuscript is IMTECH communication number 039/2018.

## Supplementary information

Supplementary Material
