# Supplementary material for: Antimicrobial properties of the novel bacterial isolate Paenibacilllus sp. SMB1 from a halo-alkaline lake in India
Source: Sci Rep. 2019 Aug 9;9:11561. doi: 10.1038/s41598-019-47879-x (PMC6688988; doi:10.1038/s41598-019-47879-x)
Supplement: Supplementary file 1 — Supplementary Material [file 41598_2019_47879_MOESM1_ESM.pdf]

Antimicrobial properties of the novel bacterial isolate *Paenibacilllus* sp. SMB1 from a halo-alkaline lake in India

Harjodh Singh[1, 2, 4, +], Manpreet Kaur [1,2,4 +], Manoj Jangra [3], Sunita Mishra[1, 2], Hemraj Nandanwar[1, 3], Anil Kumar Pinnaka[1, 4, *]

[1]Academy of Scientific and Innovative Research, (AcSIR), CSIR Campus, Chennai, India

[2] Council of Scientific and Industrial Research (CSIR) - Central Scientific Instruments Organisation, Sector 30C, Chandigarh-160030, India

[3]Clinical Microbiology & Bioactive Screening Laboratory, Council of Scientific & Industrial Research - Institute of Microbial Technology, Sector -39A, Chandigarh, India

[4]MTCC-Microbial Type Culture Collection & Gene Bank, CSIR-Institute of Microbial Technology, Chandigarh-160036, India

Address for correspondence

[*] Dr. P. Anil Kumar

MTCC-Microbial Type Culture Collection & Gene Bank, CSIR-Institute of Microbial Technology, Sector 39A, Chandigarh-160036, India

E-mail: apinnaka@imtech.res.in

Telephone: +91-172-6665728

[+] These authors contributed equally to this work.

Table S1 Phenotypic fingerprinting of the strain *Paenibacillus sambharensis* SMB1^T and strain *Paenibacillus tarimensis* DSM-19409^T using VITEK 2 is the automated system (the data in the presented study is generated under similar conditions)

VITEK (GP):	***Paenibacillus sambharensis* SMB 1^T**	***Paenibacillus tarimensis* DSM-19409^T**
D-Amygdalin	-	+
Phosphatidylinositol phospholipase C	-	-
D-Xylose	-	-
Arginine dihydrolase 1	+	-
β-Galactosidase	-	-
α-Glucosidase	+	-
Ala-Phe-Pro arylamidase	-	-
Cyclodextrin	-	-
L-Aspartate arylamidase	-	-
β-Galactopyranosidase	-	-
ALPHA-Mannosidase	-	-
Phosphatase	-	-
Leucine arylamidase	-	-
L-Proline arylamidase	-	-
β-Glucuronidase	-	-
α-Galactosidase	-	+
L-Pyrrolidonyl-arylamidase	+	+
β-Glucuronidase	-	-
Alanine arylamidase	-	-
Tyrosine arylamidase	+	-
D-Sorbitol	-	+
Urease	+	+
Polymixin B resistance	-	-
D-Galactose	-	-
D-Ribose	-	-
L-Lactatealkalinization	-	+
Lactose	-	-
N-Acetyl-D-glucosamine	-	-
D-Maltose	-	-
Bacitracin resistance	-	+
Novobiocin resistance	-	-
D-Mannitol	-	+
D-Mannose	-	+
Methyl-β-D-glucopyranoside	-	+
Pullulan	-	-
D-Raffinose	-	-
O/129 resistance (comp.vibrio.)	-	-
Salicin	-	+

Sucrose	-	+
D-Trehalose	-	+
Arginine dihydrolase 2	+	+
Optochin resistance	-	+

+, Positive; –, negative;

Table S2 Comparison of the fatty acid composition of strain *Paenibacillus sambharensis* SMB1^T with the closely related type strain *Paenibacillus tarimensis* DSM-19409^T

**Fatty acid composition**	***Paenibacillus sambharensis* SMB1^T**	***Paenibacillus tarimensis* DSM 19407^T**
**Saturated**		
C14 : 0	ND	2.73
C16 : 0	**18.17**	6.58
C17:0	4.06	ND
**Unsaturated**		
C16 : 1 *ω11c*	ND	1.86
**Branched Fatty acids**		
iso-C14 : 0	ND	2.34
iso-C15 : 0	4.17	6.66
iso-C16 : 0	5.83	5.36
iso-C17 : 0	5.09	ND
anteiso-C15 : 0	**48.86**	**61.69**
anteiso-C17 : 0	**13.82**	**12.79**

Results are shown in percentage of the total fatty acids. Fatty acids amounting to 10% or more of the total fatty acids are in bold. ND –Not Detected.

Supplementary Figure

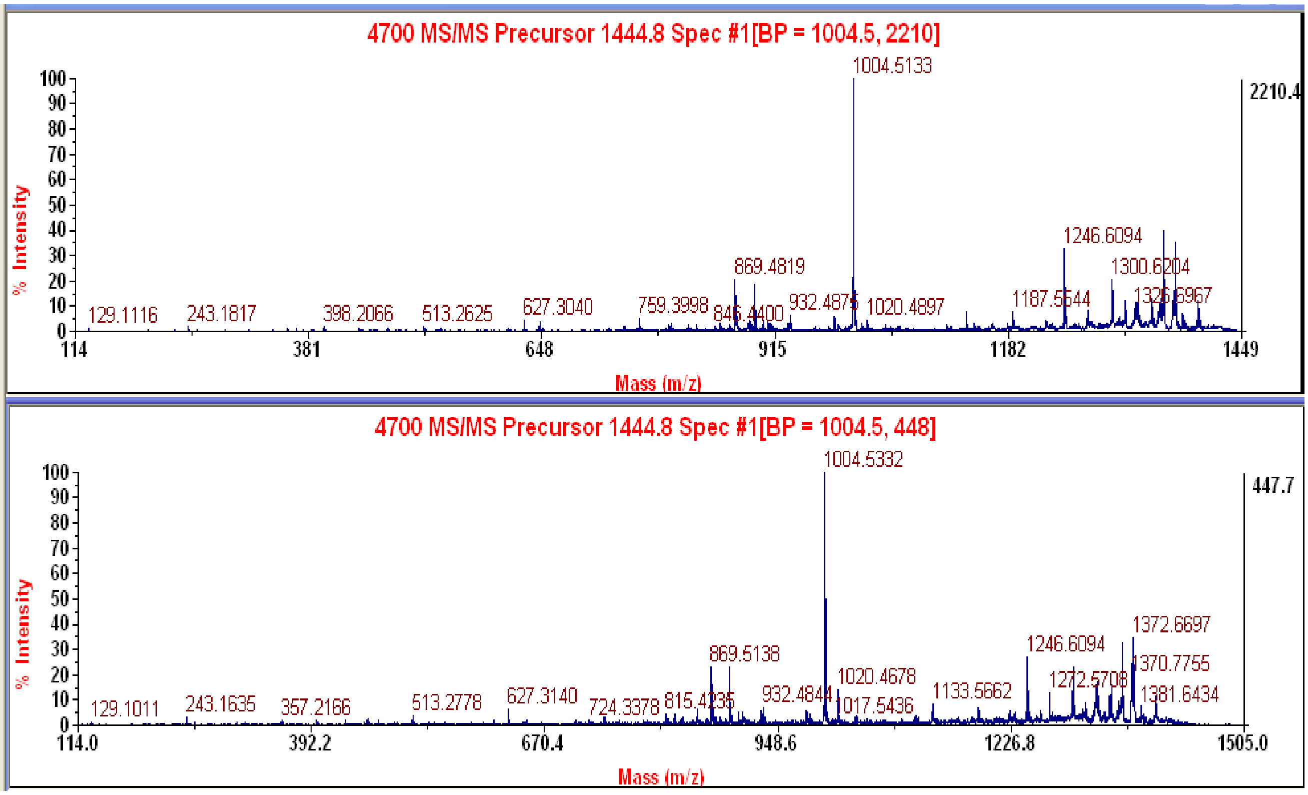

Figure S1 MSMS spectra of standard bacitracin A (top) and SMB1 peptide (bottom)

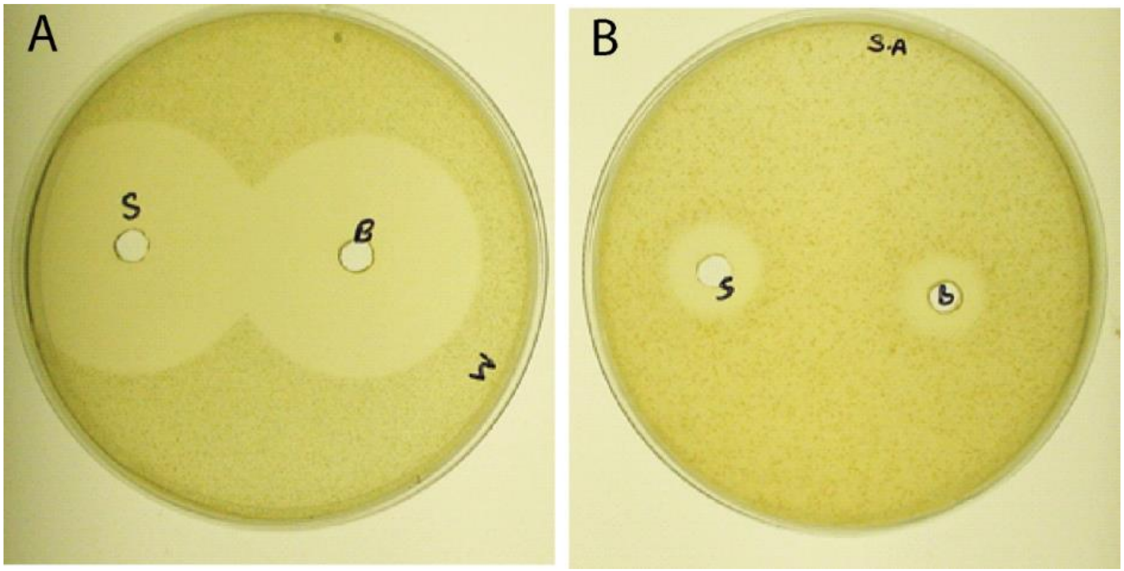

Figure S2. Purified peak from HPLC of SMB1^{T} and Bacitracin showing activity against *Micrococcus luteus* MTCC 106 and *Staphylococcus aureus* ATCC 25923. Both compounds were used at concentration 0.5mg/ml.

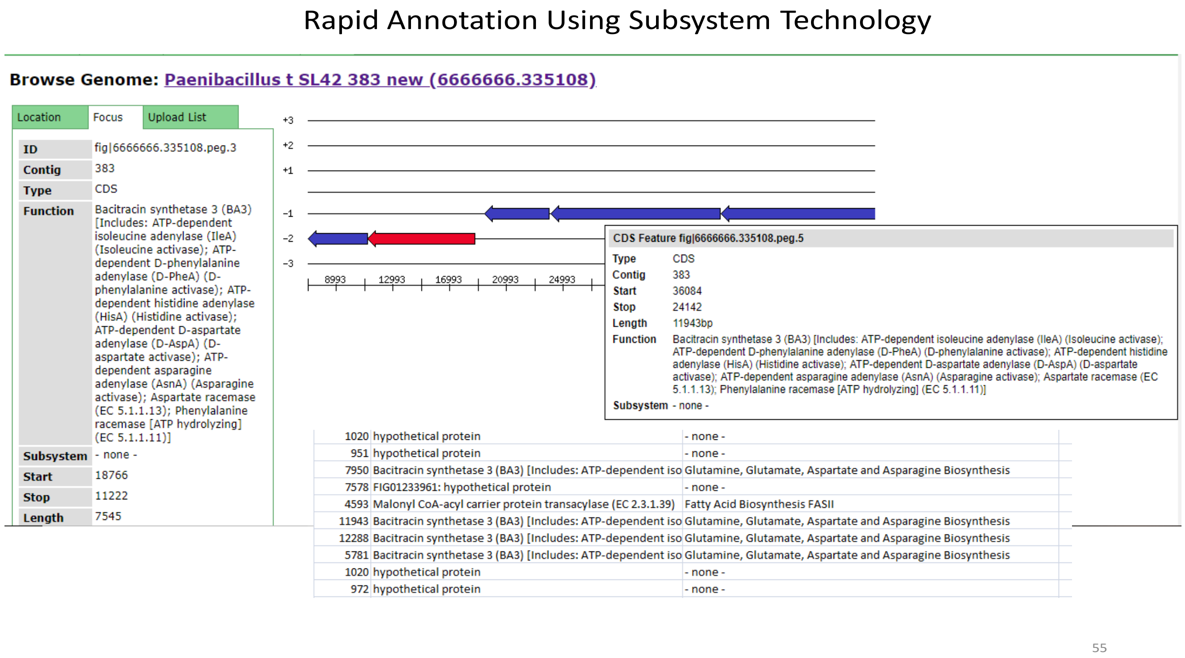

Figure S3. Rapid Annotation Using Subsystem Technology (RAST) showing the presence of Bacitracin synthetase 3 (BA3) genes [Includes: ATP-dependent isoleucine adenylase (IleA) (Isoleucine activase); ATP-dependent D-phenylalanine adenylase (D-PheA) (D-phenylalanine activase); ATP-dependent histidine adenylase (HisA) (Histidine activase); ATP-dependent D-aspartate adenylase (D-AspA) (D-aspartate activase); ATP-dependent asparagine adenylase (AsnA) (Asparagine activase); Aspartate racemase (EC 5.1.1.13); Phenylalanine racemase [ATP hydrolyzing] (EC 5.1.1.11)]

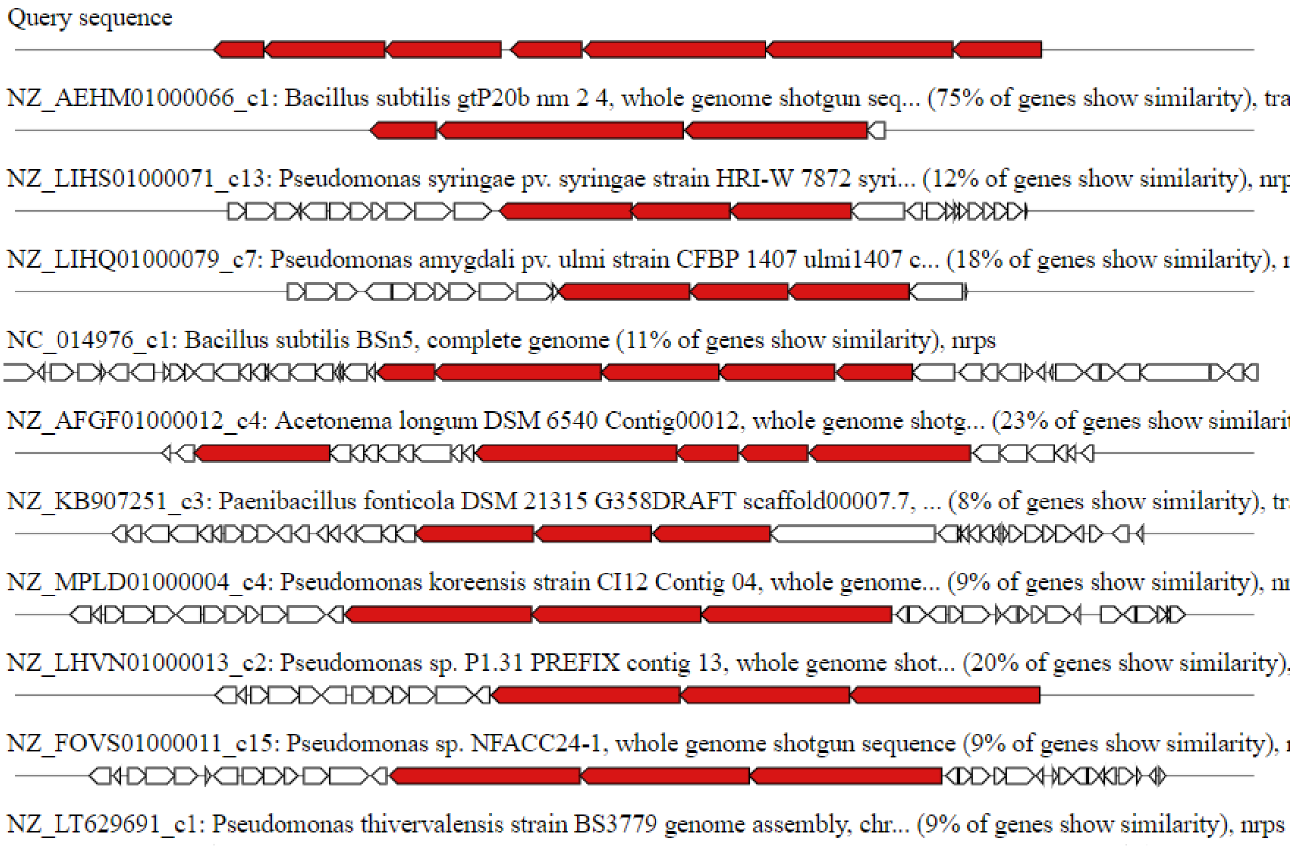

Figure S4 Comparison of NRPS gene cluster (as query sequence) present in SMB1 whole genome with other homologs.

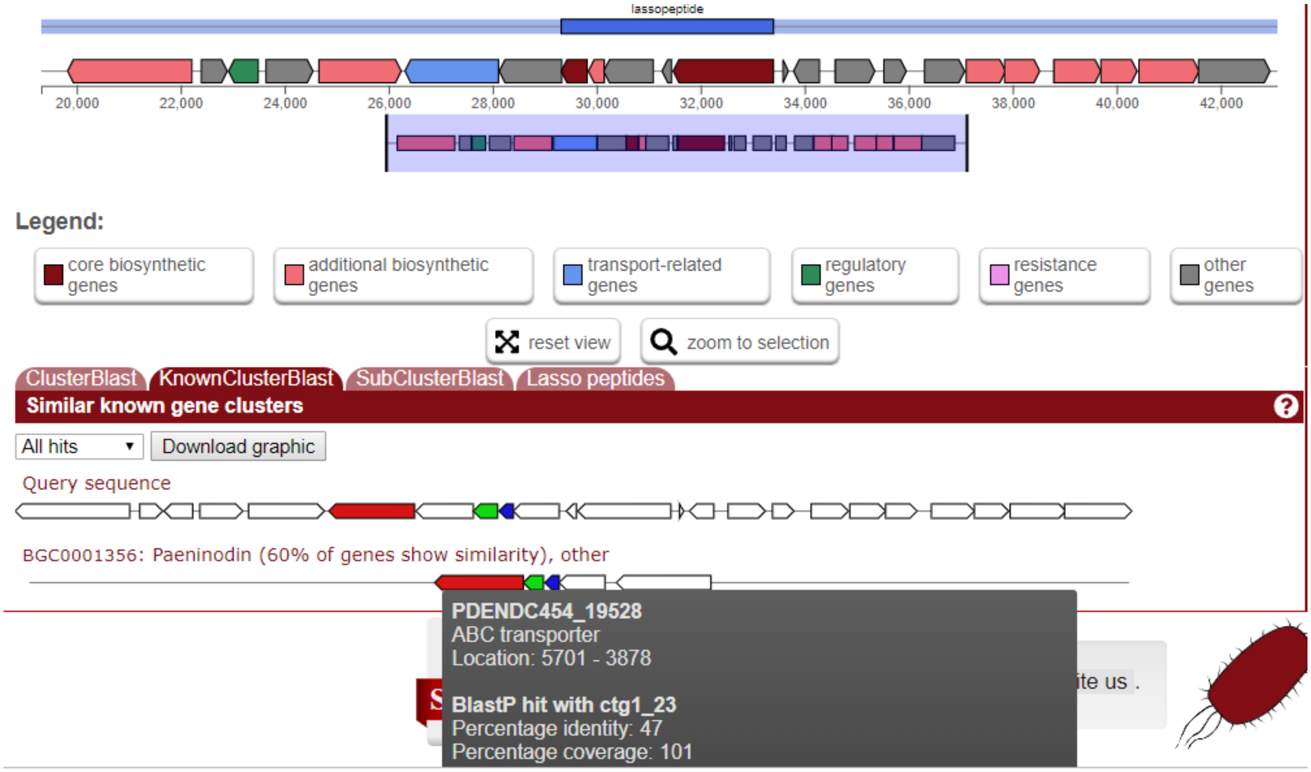

Figure S5 Comparison of SMB1 lassopeptide with its closest homolog from other bacterial species. The sequence identity was less than 40%.

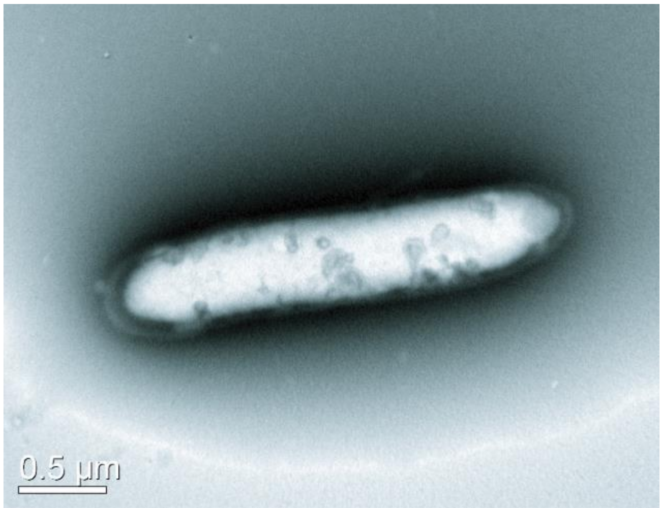

Figure S6 Electron micrograph of strain SMB1^{T}. Bar, 0.5 µm.
